# Advances in Research on the Bioactivity of Alginate Oligosaccharides

**DOI:** 10.3390/md18030144

**Published:** 2020-02-28

**Authors:** Maochen Xing, Qi Cao, Yu Wang, Han Xiao, Jiarui Zhao, Qing Zhang, Aiguo Ji, Shuliang Song

**Affiliations:** 1 Marine College, Shandong University, Weihai 264209, China; sddxxmc@163.com (M.X.); sddxcqq@163.com (Q.C.); wy392191187@163.com (Y.W.); 15651795075@163.com (H.X.); 201936684@mail.sdu.edu.cn (J.Z.); zhangqianzq@sdu.edu.cn (Q.Z.); jiaiguo@sdu.edu.cn (A.J.); 2 School of Pharmaceutical Sciences, Shandong University, Jinan 250012, China

**Keywords:** alginate, alginate oligosaccharide, biological activity, structure

## Abstract

Alginate is a natural polysaccharide present in various marine brown seaweeds. Alginate oligosaccharide (AOS) is a degradation product of alginate, which has received increasing attention due to its low molecular weight and promising biological activity. The wide-ranging biological activity of AOS is closely related to the diversity of their structures. AOS with a specific structure and distinct applications can be obtained by different methods of alginate degradation. This review focuses on recent advances in the biological activity of alginate and its derivatives, including their anti-tumor, anti-oxidative, immunoregulatory, anti-inflammatory, neuroprotective, antibacterial, hypolipidemic, antihypertensive, and hypoglycemic properties, as well as the ability to suppress obesity and promote cell proliferation and regulate plant growth. We hope that this review will provide theoretical basis and inspiration for the high-value research developments and utilization of AOS-related products.

## 1. Introduction

Alginate is a naturally occurring anionic polymer, most often isolated from the brown seaweed. It is an acidic polysaccharide belonging to the family of linear (unbranched), non-repeating copolymers. Alginate has been extensively investigated and used in many biomedical applications due to its biocompatibility, low toxicity, relatively low cost, and the ability to form a gel under mild conditions in the presence of divalent cations such as Ca ^2+^ [1].

Alginate consists of a variable amount of β-D-mannuronic acid and its C5-epimer, α-L-guluronic acid, linked by β-1,4-glycosidic bonds [2]. The monomeric composition and sequential structure of alginate vary widely between samples obtained from different species or different types of algal tissue [3]. Commercial alginates are currently extracted from marine algae such as *Laminaria* and *Macrocystis*. It can also be obtained from bacterial sources, namely *A. vinelandii* and various species of *Pseudomonas* such as *Pseudomonas aeruginosa* and *Pseudomonas mendocina* [4,5,6,7]. The difference between the two alginates is that acetylation occurs at the hydroxyl groups at position C2 and/or C3 of the mannuronic acid residue in alginate produced by bacteria [8]. In contrast to algal alginate and alginate of *A. inelandii*, the absence of G-blocks is characteristic for alginate from most bacteria such as *P. aeruginosa* [9]. Alginate is the only natural marine biopolysaccharide that contains a carboxyl group in each sugar ring. Typically, three different types of alginate polymer blocks are present: poly-α-L-guluronic acid (pG), poly-β-D-mannuronic acid (pM), and the heteropolymer of mannuronic acid and glucuronic acid (pMG) [10,11]. Although mannuronic acid (M) and glucuronic acid (G) are epimers differing only at C-5, they possess distinct conformations. In pM, all M residues assume the ^4^C_1_ conformation and are linked by β-1,4-glycosidic bond, while in pG, all G residues are in the ^1^C_4_ conformation and are linked by an α-1,4-glycosidic bond. These features are responsible for the differences in their higher-order structure. For example, pG exhibits an egg-box-like conformation and usually forms stiffer 2-fold screw helical chains when dissolved in water, while pM forms belt chains through intra-molecular hydrogen bonds. Due to these dissimilarities, pM and pG, as well as their derivatives, will exhibit different activities [12].

As the most abundant marine biomass and low-cost material, alginate has been extensively used in the food and medical industries. The widespread utilization is also driven by its favorable chemical properties and versatile activities. However, the applications of alginate have been greatly limited due to its high molecular weight and low bioavailability. Therefore, the degradation of high molecular weight polysaccharides into low molecular weight poly- or oligosaccharides is considered of great significance for improving their bioavailability, increasing the body’s absorption of drugs, and fully utilizing the efficacy of polysaccharides. Recently, the degradation products of alginate, i.e., alginate oligosaccharides (AOS), have attracted increasing attention due to their biological activities and excellent solubility in water [13]. AOS can be depolymerized by different degradation methods, including enzymatic degradation, acid hydrolysis, and oxidative degradation [14]. Alginate lyases have been isolated from a wide range of organisms, including algae, marine invertebrates, and marine and terrestrial microorganisms, which can degrade alginate into unsaturated oligosaccharides by β-elimination [15,16]. Moreover, due to differences in degradation patterns, G content (G/M ratio), molecular weight, and spatial conformation of degradation products, AOS possess a variety of biological activities. They have anti-tumor properties [17], counteract oxidation [8], regulate immune responses [18], reduce inflammation [19], are neuroprotective [20], provide antibacterial activity [21], lower lipid levels [22], reduce hypertension [23], suppress obesity [24], decrease blood sugar levels [25], promote cellular proliferation and regulate plant growth [26]. Due to these properties, AOS have found a wide range of applications in the agricultural, food, and pharmaceutical industries [27].

This review focuses on recent advances in the research on alginate, AOS, and their derivatives, including their biological activities, mechanisms of action, and factors that affect their activity. The objective is to provide a theoretical basis for further development and utilization of alginate.

## 2. Biological Activity of Alginate Oligosaccharides

### 2.1. Anti-Tumor Activity

Cancer is the leading cause of death in economically developed countries and the second most frequent cause of death in developing countries [28]. Chemotherapy has long been an important modality of cancer treatment [29] but is often accompanied by severe adverse effects [30]. For example, the platinum-based drugs cisplatin, carboplatin, and oxaliplatin are routinely prescribed for cancer treatment but, while they are effective, their use is limited by severe, dose-limiting side effects [31]. To solve the problem of toxicity of available chemotherapeutic agents, a growing number of scientists are searching for non-toxic anti-tumor natural products in the ocean. Amongst these, AOS has become an attractive candidate for biomedical applications as a nonimmunogenic, non-toxic and biodegradable polymer [32].

The anti-tumor effects of AOS involve a variety of mechanisms, including inhibition of proliferation and migration of tumor cells, regulation of immune defense responses, and improvement of antioxidant and anti-inflammatory capabilities. For example, AOS has been demonstrated to attenuate the proliferation, migration, and invasion of human prostate cancer cells through the suppression of the Hippo/YAP/c-Jun pathway [17]. In addition, there is increasing evidence that AOS achieves its anti-tumor effects through immunomodulation. AOS, such as enzymatically depolymerized guluronate and mannuronate oligomers (enzymatic degradation, degree of polymerization: 20–24, concentration: 500 μg/mL) enhance defense mechanisms against human leukemia cells U937 by upregulating the synthesis cytotoxic cytokines in human mononuclear cells, and these effects are inhibited by antibodies to tumor necrosis factor (TNF) -α [33]. AOS derivatives exert significant anti-tumor activity by immunomodulation. Sulfated derivatives of AOS (molecular weight: 3798 Da, sulfation degree: 1.3, concentration: 100 μg/kg) induce tumor regression indirectly by modulating immune responses of the host [34]. Polyalcoholized mannoglucan (MGA, molecular weight: 300–1000 kDa, concentration: 100 μg/mouse) selectively induces the TNF-like cytotoxic factor in tumor tissues [35], providing local therapeutic effect on MH134 hepatoma in mice. Importantly, the almost complete regression of tumors induced by MGA was accompanied by only minimal local inflammatory reaction. These findings indicate that the anti-tumor mechanism of this form of alginate MGA is not mediated merely by accumulation of active inflammatory cells within the tumor, but by other activities, which need to be further clarified [36]. The local anti-tumor activity may be explained by site-specific stimulation of effector cells since a degraded d-manno-d-glucan (DMG) exhibits this property, and untreated MH134 tumor tissue contains infiltrating host cells [37].

Cancer-related inflammation (CRI) plays a critical role in tumor progression, angiogenesis, metastasis, and immunosuppression. Therefore, improving the anti-oxidative and anti-inflammatory potential of the organism by AOS may constitute another mechanism by which these molecules can protect against cancer. AOS (molecular weight: 1009 Da, quantities ingested: oral administration of 10-mg AOS daily) prepared by enzymatic hydrolysis has been shown to reduce the progression of osteosarcoma in a concentration-dependent manner, and this effect was associated with an improvement in antioxidant and anti-inflammatory capacities of the patients [29].

Research shows that α-l-guluronic acid (ALG), a potent anti-inflammatory agent on breast cancer-related inflammation both in vitro and in vivo conditions, can effectively inhibit the CRI and tumor-promoting mediators (Cyclooxygenase-2, (COX-2), matrix metalloproteinase2, (MMP2), matrix metalloproteinase9 (MMP9), vascular endothelial growth factor (VEGF) and proinflammatory cytokines) without direct toxic effects on the cells. Moreover, it was found that ALG (concentration: 50 mg/kg (therapeutic group)) can effectively inhibit the tumor cell adhesion to extracellular matrix, seeding in implantation tissue, and reduce accumulation of immunosuppressive and inflammatory cells in tumor-bearing mice. These findings were associated with decreased tumor growth, metastasis, angiogenesis and prolonged mice survival. These findings could lead to the establishment of novel non-steroidal anti-inflammatory drugs (NSAIDs)-based cancer therapy in the near future and open a new horizon for cancer treatment [1].

Current research on the anti-tumor activity of alginate and alginate-derived oligosaccharides is focused predominantly on eliminating tumor cells by immunomodulation and anti-inflammatory ability. Despite the identification of a variety of anti-tumor properties of AOS, the in-depth research of their mechanism is limited, restricting further development and utilization of AOS in cancer treatment.

The anti-tumor activity of AOS can be affected by multiple factors, such as G content, conformation, viscosity, and substituent groups. In AOS of comparable molecular weight, the presence of higher levels of MM-blocks may be associated with higher anti-cancer potency [38,39]. This difference in activity may be the consequence of distinct conformations of d-mannuronan and l-guluronan. This notion is supported by the finding that after the addition of 3 mM Ca^2+^ to the alginate with high GG-block content, the circular dichroism spectrum of the alginate approximated that of poly-d-mannuronate with a high MM-block content. At this time, the alginate with lower MM-block content can also exhibit high anti-cancer potency [39]. Therefore, the anti-tumor activity of alginate may depend on its conformation, which is closely related to the G content [38].

A study was performed on the in vivo activity of two brown seaweed alginates, the high- viscosity alginate and the low-viscosity alginate, against sarcoma 180 cells implanted in mice. At the same dose, the high-viscosity alginate exhibited a stronger inhibition of sarcoma 180 cell growth than the low-viscosity alginate [39]. Interestingly, the low-viscosity alginate has a higher M/G ratio than the high-viscosity alginate, which is not consistent with previous studies demonstrating that a high proportion of MM-blocks is associated with a stronger anti-tumor activity [33]. These results suggest that G content in alginate may affect its anti-tumor activity to a certain extent, but is not the exclusive factor determining this property. In addition, the sulfated derivative of AOS exhibits better anti-tumor activity than AOS [34]. This difference may reflect the presence of sulfate groups, which improve the solubility of AOS and increase its charge density. Higher charge density is associated with an increase in the polarity of AOS molecules, ultimately facilitating the interaction between sulfated AOS and cell surface [40]. In conclusion, the structure of AOS has a profound impact on its anti-tumor activity. However, it should be realized that a single structural modification of AOS cannot fully determine the impact on its overall anti-tumor activity. Ultimately, the activity of AOS is determined by multiple factors such as AOS composition, conformation, and substituent groups.

### 2.2. Antioxidant Activity

Reactive oxygen species (ROS) are products of normal metabolism and xenobiotic exposure [41], such as superoxide (•O^2−^), hydrogen peroxide (H_2_O_2_), organic peroxides (ROOR′), hydroxyl radical (OH•), and peroxynitrite (ONOO^−^) [42]. ROS participate in intracellular signaling cascades and are required in some biological reactions. However, excessive formation of ROS may lead to harmful oxidative stress, which may induce structural and molecular damage in the cell [43]. Intracellular generation of reactive oxygen intermediates threatens the integrity of various biomolecules including proteins [44], DNA [45], and lipids and lipoproteins involved in atherosclerosis [46]. Excessive oxidative damage results in loss of essential cell functions, eventually leading to apoptosis or necrosis [47]. Scavenging of ROS or inhibition of their production by natural or synthetic antioxidants can effectively prevent oxidative stress-induced cell death. Many synthetic chemicals such as phenolic compounds have been proven to act as potent free radical scavengers, but they often produce severe side effects [48]. Therefore, attention has been focused recently on identification of natural substances that can scavenge free radicals and protect cells from oxidative damage [49]. 

**Figure 1:**
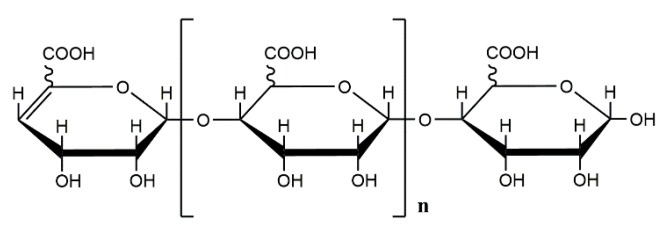
Schematic representation of the molecular structure of alginate oligosaccharide prepared by enzymatic degradation [50].

Several studies have documented that AOS effectively inhibits oxidative stress, affording an adequate resistance to diseases caused by oxidative stress. AOS generated by enzymatic degradation (concentration: 1% (w/v)) not only effectively prevents oxidative stress-induced neurotoxicity, but also inhibits the formation of amyloid β in an in vitro model of Alzheimer’s disease. This cytoprotective effect of AOS is dependent on the upregulation of heme oxygenase-1 and γ-glutamylcysteine synthetase in the NF-E2-related factor 2 pathway [20]. Pretreatment with AOS (enzymatic degradation, molecular weight: 1.2 KDa, degree of polymerization: 2-6, M/G ratio 1/2.6, concentration: 200 mg/kg/day, 7 days) suppressed oxidative stress by downregulating the expression of gp91-phox (also named NADPH oxidase2, (NOX2)) and 4-hydroxynonenal (4-HNE) protein in the heart, preventing acute doxorubicin cardiotoxicity [14]. Similarly, the pretreatment with AOS (enzymatic degradation, concentration: 200 mg/kg/d, 7 days) suppressed the upregulation of NOX2 and 4-HNE, which are responsible for excessive oxidative stress in acute myocardial ischemia/reperfusion injury in mice (Figure 1) [50]. These studies indicate that AOS is an antioxidant with potential clinical applications, and the pharmacokinetics of AOS and mechanism of its antioxidant activity may become another challenging area of research.

Several factors influence the antioxidant properties of AOS, including concentration, molecular weight, and depolymerization methods. Low molecular weight alginate shows scavenging activity against ABTS and superoxide radicals in a concentration- and time-dependent manner, and this activity is inversely correlated to the molecular weight of alginate [51]. AOS (enzymatic degradation) with a molecular weight of less than 1 kDa are better scavengers of superoxide, hydroxyl, and hypochlorous acid free radicals than alginate with a molecular weight of 1–10 kDa, ascorbic acid, and carnosine. In addition, the AOS with molecular weight of 1–6 kDa have higher antioxidant activity against superoxide and hypochlorous acid free radicals than AOS with molecular weight of 6–10 kDa, even if they have a similar M/G ratio [52]. Thus, low molecular weight AOS exert higher antioxidant activity.

Surprisingly, the antioxidant activity is independent of the relative content of mannuronate and guluronate in AOS. Given that the only structural difference between pM and pG is the orientation of the C5 carboxyl groups, their spatial arrangement does not affect the antioxidant activity of alginate [53]. The depolymerization method also influences the antioxidant mechanism and activity of alginate. The antioxidant mechanism of alginate possibly involves the scavenging of radicals by hydrogen abstraction from the carbon-bonded hydrogen atoms [54]. The antioxidant activity and mechanism of action of pG and pM prepared by acid hydrolysis are comparable to those of polymeric alginate. In contrast, preparation of AOS by enzymatic hydrolysis is accompanied by the formation of a conjugated alkene acid structure. Therefore, AOS obtained by enzymatic hydrolysis shows higher antioxidant activity than the polymeric and monomeric forms of alginate and acid-hydrolyzed alginate, which lack a conjugated enoic acid structure [53].

Currently, although the ability of AOS to scavenge free radicals is well-documented experimentally, there are only a few studies on its mechanism. The knowledge of the molecular basis of the antioxidant activity will allow for rational modifications of AOS, tailoring the molecules towards specific applications. These developments can be expected to increase the use of AOS in the food and pharmaceutical industries.

### 2.3. Immunoregulatory Activity

An increasing body of evidence shows that natural polysaccharides and oligosaccharides have the potential to act as immunomodulators. One of the key functions of alginate and AOS in immunoregulation, addressed by several studies, is the induction of cytokine activity [18]. AOS (enzymatic degradation, degree of polymerization: 3–6, concentration: 500 μg/mL) can induce the synthesis of cytokines in RAW264.7 macrophages. TNF-α, granulocyte colony-stimulating factor (GCSF), monocyte chemoattractant protein-1 (MCP-1), Regulated on Activation, Normal T Expressed and Secreted (RANTES), granulocyte-macrophage colony-stimulating factor (GM-CSF), and eotaxin can all be induced by AOS to a different extent, depending on the oligomer structure. Additionally, AOS activate also the formation of low but significant levels of interleukin (IL)-1α, IL-1β, IL-6, IL-9, and IL-13 [55]. AOS (enzymatic degradation, degree of polymerization: 3–9, molecular weight: 529–1585 Da, concentration: 70 mg/kg (intraperitoneal administration)) can also activate the synthesis of multiple immunoreactive cytokines in mice, including GCSF, MCP-1, IL-6, keratinocyte-derived chemokine (KC), IL-12, RANTES [56,57]. Together, these results document the ability of AOS to activate macrophages and the host immune system [58]. Importantly, the anti-tumor and immunomodulatory effects of alginate are inseparable. For example, sodium alginate (molecular weight: 9500 kDa, M/G 1.96, concentration: 3 mg/mL) can induce RAW264.7 macrophages to produce cytokines, such as IL-1b, IL-6, IL-12 and TNF-α, via the nuclear transcription factor-κB (NF-κB) signaling pathway [59] and can exert anti-tumor activity against the sarcoma 180 solid tumor in mice by the activation of the host immune system [60]. The latter aspect of alginate activity has been elaborated under Section 2.1.

In addition, research efforts have been focused on the macrophage immuno-inflammatory responses induced by unsaturated guluronate oligosaccharide (GOS) derived from pG. For example, enzymatically depolymerized unsaturated alginate, such as GOS (concentration: 1000 μg/mL), can induce tumor necrosis factor and reactive oxygen species secretion from RAW264.7 cells, and it can exert immunomodulatory effects by increasing the expression of inducible nitric oxide synthase (iNOS) to increase the production of nitric oxide (NO). Therefore, GOS is considered to be an effective activator of macrophages [61].

The toll-like receptor (TLR) 4-related protein kinase B (Akt)/NF-κB and Akt/mechanistic target of rapamycin (mTOR) pathways are essential for the activation of macrophages by GOS and its immunostimulatory activity. The immune regulation in RAW264.7 macrophages mediated by GOS (enzymatic degradation, degree of polymerization: 2–8, concentration: 1 mg/mL) can be divided into the following main steps: (1) The surface receptor TLR4 recognizes GOS; (2) phosphatidylinositol-3-kinase (PI3K) is activated by TLR4 and subsequently induces the phosphorylation of Akt; (3) phosphorylated Akt triggers I-κB phosphorylation, resulting in (4) the release and translocation of NF-κB into the nucleus; (5) mTOR and p70 S6 kinase (p70 S6K) are also activated by phosphorylated Akt; (6) all these factors work together or separately to activate the synthesis of inflammatory mediators [18,33,57,62,63].

**Figure 2:**
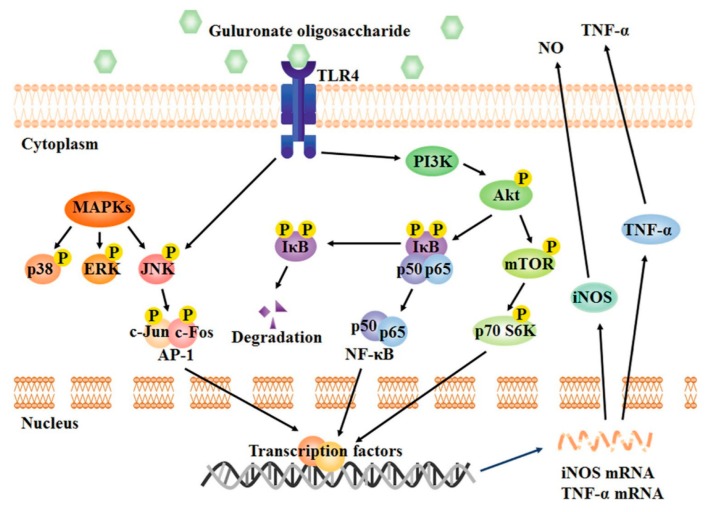
Signaling pathways involved in the macrophage activation effect of alginate-derived guluronate oligosaccharide [18].

One of the most important pathways downstream of TLR4 is the mitogen-activated protein kinases (MAPKs) signaling pathway [61,64]. GOS increases the phosphorylation of MAPKs and activates all components of the MAPK pathway, including p38, c-Jun N-terminal kinase (JNK) and extracellular signal-regulated kinase (ERK) (Figure 2) [18,57]. These events trigger the activation of downstream transcription factors and, consequently, initiation of transcription of genes controlling the synthesis of NO and TNF-α. However, it should be noted that only the phosphorylation of JNK depends on the binding of GOS to the TLR4 receptor; p38 and ERK are not affected by TLR4. Thus, TLR4 is not the only receptor by which GOS can activate macrophages. In this regard, it has been documented that GOS (concentration: 1 mg/mL) can also upregulate the expression of Fcγ receptor (FcγR)I and FcγRII on the macrophage surface, resulting in increased phagocytosis capacity [58]. Moreover, GOS activates also TLR2 in RAW264.7 cells [61]. Further research is required to discover additional receptors of GOS on macrophages and elucidate completely the mechanism of GOS-induced immune responses in macrophages.

Overall, it should be recognized that the effect of GOS on RAW264.7 macrophages is not restricted to a single pathway, but that it can activate both the pro-inflammatory and anti-inflammatory responses in these cells. Proteomics studies have found that after the GOS (degree of polymerization: 2–8, concentration: 1 mg/mL) treatment of RAW264.7 cells, differentially expressed proteins are involved in the functional pathways of activation and suppression of the NF-κB signaling, pro- and anti-inflammatory pathways, pro- and anti-oxidation mechanisms, cytoskeletal remodeling, and cell proliferation. All these regulatory effects of GOS can improve macrophage homeostasis. For example, the levels of galectin-1 (LGALS1) and lactoylglutathione lyase (GLO1), two proteins involved in the NF-κB pathway, were increased after GOS treatment of macrophages. Conversely, the upregulation of expression of LGALS1 and GLO1 prevents the excessive activation of NF-κB signaling in GOS-treated cells, suggesting the presence of a negative feedback regulatory loop responsible for the maintenance of cell homeostasis [65]. 

It should not be overlooked that α-l-guluronic acid monosaccharide also shows relatively high immunomodulatory activity. α-l-guluronic acid (concentration: 5 μg/mL) can significantly reduce the expression of the TLR2 and NF-κB genes in peripheral blood mononuclear cells in patients with common variant immunodeficiency (CVID), facilitating the control of autoimmune presentation [66]. Additionally, α-l-guluronic acid (concentration: 25 μg/mL) exerts its immunomodulatory effects by the downregulation of NF-κB, I-κB, and myeloid differentiation factor 88 gene expression and suppressing the synthesis of IL-1β. These effects of α-l-guluronic acid are dose-dependent and are mediated by the toll-interacting protein [67]. The above-presented studies expand our understanding of the role of alginate in immune regulation and broaden the range of potential clinical applications of alginate as an immunomodulatory agent, including its use in cancer therapy.

**Figure 3:**
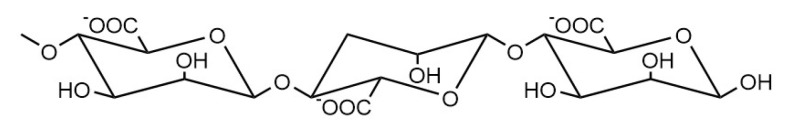
Schematic representation of the molecular structure of saturated mannuronate oligomers prepared by acid hydrolysis [61].

The structural features of AOS, especially its unsaturated terminal structure and composition, are essential for the activation of RAW264.7 macrophages [57]. At present, a growing number of investigations are focused on the relationship between the unsaturated terminal structure and activity of AOS [58]. The formation of oligomers by enzymatic hydrolysis of alginate is essential for some of the biological effects of AOS on human monocytes. Enzymatically depolymerized unsaturated alginate oligosaccharide can induce the secretion of TNF-α by RAW264.7 cells, and this effect is structure-dependent. In contrast, the activity of the saturated alginate oligosaccharide prepared by acid hydrolysis is low. Thus, the presence of the unsaturated terminal structure in AOS obtained by enzymatic degradation is essential for the activation of macrophages [33,55,61]. These results indicate that both plants and mammals have a common recognition mechanism capable of distinguishing the saturated and unsaturated terminal structure of AOS (Figure 3) [61]. Therefore, a variety of alginate lyases could be used in the future to produce unsaturated AOS and develop more functional alginates suitable for industrial applications.

The composition of AOS is an additional variable determining the effects of AOS on the synthesis of cytokines by macrophages, but some controversies continue to exist in this research area. It has been shown that the cytotoxicity of conditioned medium prepared from mononuclear cells cultured with guluronate oligomers is somewhat higher than that of cells treated with mannuronate oligomers, suggesting that guluronate oligomers are slightly more potent activators of mononuclear cells than mannuronate oligomers [33]. Interestingly, another study documented that mannuronate oligomers tend to be more effective inducers of cytokines than guluronate oligomers [55]. This discrepancy may reflect the role of other factors, besides the composition, such as molecular weight and conformation of the molecule, that affect the AOS activity.

In summary, AOS is a promising natural additive from seaweed that may be utilized by the pharmacological industry to enhance innate immunity and improve host defense against bacterial infections, and to treat autoimmune inflammatory diseases. 

### 2.4. Anti-Inflammatory Activity

An increasing amount of evidence documents that inflammation is associated with the development of several pathologic conditions, such as cancer, obesity, and cardiovascular disease [68,69]. Toll-like receptors, and TLR4 in particular, have an important function in the molecular mechanisms of inflammation [70]. The stimulation of TLR4 signaling pathway by lipopolysaccharide (LPS) triggers a cascade of pro-inflammatory events that lead to the activation of the transcription factor NF-κB, several kinases such as MAPK, Akt, and PI3K, increased synthesis of inflammation-related mediators, including NO, prostaglandin E_2_ (PGE_2_), ROS, pro-inflammatory proteins, such as iNOS and COX-2, and pro-inflammatory cytokines [71,72]. However, in most instances, excessive inflammatory reactions have adverse effects on the body.

Therefore, the anti-inflammatory activity of natural polysaccharides has been intensely investigated to identify anti-inflammatory agents acting by suppressing the production of NO and PGE_2_ and downregulating the expression of iNOS and COX-2. However, extensive application of these polysaccharides is hindered by their high molecular weight and low solubility [73,74,75]. These obstacles can be overcome by degrading the polysaccharide molecule [76]. The guluronate oligosaccharide obtained by oxidative degradation of alginate (concentration: 1 mg/mL) suppresses in a dose-dependent manner the overproduction of inflammatory mediators NO, PGE_2_, and ROS, inflammatory proteins iNOS and COX-2, and pro-inflammatory cytokines TNF-α, IL-1β, and IL-6 in LPS-activated RAW264.7 macrophages. The anti-inflammatory activity mechanism of guluronate oligosaccharides prepared by oxidative degradation (GOS-OD) may involve the inhibition of the binding of LPS to the cell surface and attenuation of LPS-induced expression of TLR4 and cluster of differentiation 14. These changes, in turn, can inhibit the activation of NF-κB and MAPK signaling pathways [77]. Similarly, the treatment of BV2 cells by AOS (oxidative degradation, molecular weight: 1500 Da, concentration: 50–500 μg/mL) markedly inhibits the LPS-activated synthesis and secretion of TNF-α, IL-6, and IL-1β, as well as the amyloid β-protein (Aβ)-activated production of TNF-α, IL-6, and IL-12. These results suggest that alginate-derived oligosaccharide (AdO) may find application in the treatment of neuroinflammation by suppressing the activation of microglia and attenuating the production of inflammatory mediators [19].

α-l-guluronic acid and β-d-mannuronic acid, the two monosaccharides obtained by degradation of alginate also show excellent anti-inflammatory activity and can be applied as effective anti-inflammatory agents. Guluronic acid (quantities ingested: 500 mg, twice a day), used as a novel NSAID, significantly reduces symptoms in ankylosing spondylitis (AS) patients within 12 weeks and also improved the clinical parameters of AS, including the severity of the disease, inflammation, and physical activity of the patients [78]. Numerous studies have shown the immunosuppressive properties of β-d-mannuronic acid (concentration: 30 mg/kg (intraperitoneal administration)) in several experimental models such as adjuvant-induced arthritis (AIA), experimental autoimmune encephalomyelitis (EAE), nephrotic syndrome and glomerulonephritis. Its therapeutic effects were characterized by excellent tolerability, safety of use, and efficacy [79,80,81]. It has been demonstrated that the anti-inflammatory activity of α-l-guluronic acid and β-d-mannuronic acid is linked to the regulation of TLR4 signaling pathway during inflammation [82,83,84].

**Figure 4:**
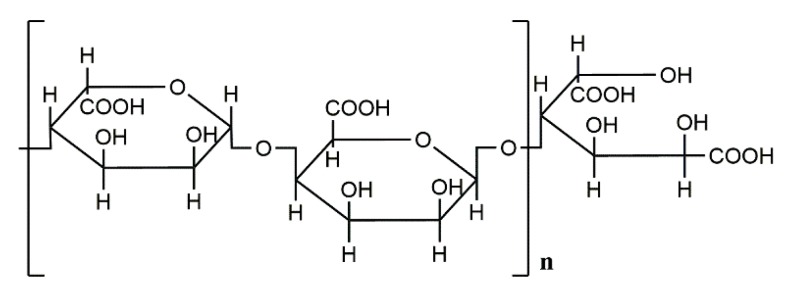
Schematic representation of chemical structures of alginate-derived oligosaccharide prepared by oxidative degradation [19]. (The average molecular weight of this AOS is about 1500 Da).

The anti-inflammatory activities of AOS obtained by different degradation methods are remarkably different. AOS prepared by oxidative degradation inhibits the inflammatory response in LPS-activated RAW264.7 macrophage, while GOS obtained by enzymatic hydrolysis and acidolysis had no such activity. In addition, AOS prepared by oxidative degradation inhibit microglial neuroinflammation stimulated by LPS and Aβ (Figure 4) [19]. These findings indicate that AOS generated by oxidative degradation is a stronger inhibitor of inflammation than AOS prepared using other methods. The structure of AOS also determines their activity. In GOS-OD, the presence of a carboxyl group at the 1-position of the reducing end affects its inhibitory effect on NO production stimulated by LPS [77]. Therefore, the influence of the degradation method on the AOS structure must be fully considered in the development of AOS with anti-inflammatory activity. The alginate obtained by the oxidative degradation method may bring novel prospects to the clinical applications of the anti-inflammatory properties of AOS.

### 2.5. Neuroprotective Activity

Cell damage induced by oxidative stress has long been associated with the ageing process and various neurodegenerative diseases, such as Alzheimer’s disease (AD) [85,86]. AOS (enzymatic degradation) not only effectively prevent neurotoxicity induced by oxidative stress and protect NT2 neurons from death, but also inhibit Aβ formation in an in vitro model of AD. The underlying mechanisms have been discussed in Section 2.2. The significant antioxidant capacity of alginate led to the conclusion that the inhibitory action of alginate on H_2_O_2_-induced formation of Aβ is likely associated with the ROS-scavenging abilities of alginate [20]. 

Several studies demonstrated that ROS promote apoptosis by activating caspase-3 activity [87]. The mutual relationship between caspase-3 activation and ROS generation raises the possibility that the inhibition of caspase-3 by alginate might be related to its ROS-scavenging property. If this were the case, the inhibition of oxidative stress-induced neurodegenerative diseases by AOS may also be due to decreasing the level of ROS. AOS (enzymatic degradation, molecular weight: 1300 Da, quantities ingested: (360 mg/kg/day, oral administration)) bearing mannuronate-rich blocks alleviate Alzheimer-type behavioral symptoms induced by scopolamine and Aβ (1–40) in rodents. In vitro studies utilizing in human neuroblastoma SH-SY5Y cells suggested that the mechanism of action of AOS may involve a reduction in the intracellular free calcium overload, resulting in the inhibition of the production of ROS and suppression of apoptosis. In addition, the binding of AOS to Aβ and subsequent inhibition of fibril formation may also contribute to its neuroprotective activity [88,89]. AOS can be found in the cerebrospinal fluid after both oral and intravenous administration, indicating that this molecule can cross the blood-brain barrier (BBB). The permeability of BBB to AOS highlights the potential for clinical use of AOS to treat neuropathological changes induced by AD [90].

It is well-documented that neuroinflammation is involved in the pathogenesis and progression of various neurodegenerative disorders, including AD [91,92] and Parkinson’s disease [93]. The accumulation of Aβ and the resulting neurotoxicity is considered to contribute significantly to the etiology of AD. The remarkable inhibition of neuroinflammation and promotion of microglial phagocytosis of Aβ by AOS (oxidative degradation, molecular weight: 1500 Da) was detailed in Section 2.4. Paradoxically, drugs that attenuate TLR signaling and reduce the inflammatory response of activated microglia are beneficial in the treatment of AD [94,95]. However, studies in mice expressing a mutated form of TLR4 demonstrated that the activation of microglial TLR4 reduces the accumulation of Aβ [96]. Therefore, TLR4 signaling can have neurotoxic or neuroprotective effects, depending on the pathological conditions. Together, the available results support the notion that TLR4 activation is tightly controlled to regulate its distinct function in neuroinflammation and microglial phagocytosis [19].

Advanced glycation end-products (AGEs) participate in the pathogenesis of AD by receptor- and non-receptor-mediated pathways and are associated with abnormal brain protein cross-linking, oxidative stress, and neuronal loss [97,98,99,100]. Naturally occurring antiglycation agents offer a novel approach to the prevention and/or treatment of diseases involving the formation of AGEs, e.g., AD, diabetes, and atherosclerosis [101]. Importantly, alginate has been recently reported as an antiglycation agent [102] and may represent a new compound to be used in the treatment of AD [100].

The past decades have witnessed repeated failures of strategies targeting Aβ or the tau protein in clinical trials of therapies for late-stage AD, necessitating the development of new approaches to the treatment of this complex disease. A recent investigation has shown that GV-971, a sodium oligomannate, produced an evident and consistent improvement of cognition in phase 3 clinical trial in China. GV-971(degree of polymerization: 2–10, molecular weight: up to 1kDa, quantities ingested: 100 mg/kg/day, oral administration) suppressed gut dysbiosis and the associated phenylalanine/isoleucine accumulation, harnessed neuroinflammation, and reversed the cognition impairment in mild-to-moderate AD patients [103]. All these findings offered the possibility of a conceptual advancement in the understanding of the etiology of AD. The new concept considers AD as not only an Aβ-driven brain disease but a consequence of systemic interaction between the gut and the brain, mediated by inflammatory factors.

It should be noted that the treatment of Alzheimer’s disease with oligo-guluronic acid (pG), an epimer of GV-971, or with polymannuronate sulfate, polyguluronate sulfate, heparin, or heparan sulfate did not show the therapeutic effects. Thus, the sugar backbone of GV-971 may account for its specific impact on gut microbiota. This effect may reflect conformational differences between pM and pG; pG exhibits an egg-box-like conformation, while pM forms belt chains through intra-molecular hydrogen bonds [104]. An in-depth study of the structure-activity relationship in AOS will provide a better understanding of the mechanism by which pM regulates the intestinal flora. 

In summary, AdO is an effective drug for the treatment of neurodegenerative diseases. In particular, the microbiota-centric anti-AD effect of GV-971 will open a new therapeutic avenue for the treatment of AD by remodeling gut microbiota, and guide the future development of effective therapies by exploring the enormous potential of complex carbohydrates. However, the mechanism responsible for the neuroprotective function of alginate remains to be further explored. Future studies identifying the crucial strains of bacteria accounting for the effects of pM on metabolic and neuroinflammation changes will be critical. In addition, extensive research will be required to identify other mechanistic links connecting gut microbiota and neuroinflammation [20,103], and uncover other possible effects of AOS on intestinal microbes.

### 2.6. Antibacterial Activity

When embedded within biofilm structures, bacteria exhibit up to 1000-fold enhanced resistance to conventional antibiotics [105,106]. Several therapeutic strategies aiming at the disruption of the biofilm have been developed but, thus far, they failed to deliver the expected results [107]. Therefore, a large unmet clinical need exists for effective anti-biofilm therapies applicable in human disease. An exciting development in this field is the demonstration that the oligosaccharide (OligoG), alginate derived from seaweed consisting of 96% of α-l-guluronic acid and in 4% of the β-d-mannuronic acid isomer, possesses antibacterial and anti-biofilm properties. OligoG potentiates the activity of certain antibiotics against multi-drug resistant bacteria [21,108]. OligoG (degree of polymerization: 16, concentration: 2% (w/v (incubation broth)) with G content greater than 85% inhibits the growth of gram-negative bacteria and synergistically enhances the activity of colistin against *P. aeruginosa*, strain NH57388A, benefiting cystic fibrosis patients treated with this antibiotic [107,109]. A recent study in a murine model of lung infection confirmed that OligoG (concentration: 5% (m/v (0.9% saline)) containing more than 85% of α-l-guluronic acid and less than 15% of β-d-mannuronic acid can prevent the formation of biofilm formation in a dose-dependent manner [110]. Increasing evidence shows that capacity of OligoG to overcome the resistance of bacteria and biofilms depends on a variety of mechanisms, most of which involve the destruction of the network of extracellular polymeric substance (EPS) of the biofilm. Bacterial EPS consists mainly of polysaccharides, proteins, lipids, RNA, and extracellular DNA, and facilitates the formation and maturation of the biofilm [111]. Considering its importance in maintaining biofilm structure and physiology, numerous strategies have been developed to disrupt EPS disruption. The most recent findings are discussed below.

OligoG CF-5/20 (Oligo G) sourced from the brown seaweed can readily diffuse into established biofilms, modify their EPS, disrupt DNA-Ca^2+^-DNA bridges and biofilm EPS matrix, interfere with the formation of pseudomonal microcolonies, and potentiate antibiotic activity [107]. Similarly, in combination therapies of respiratory diseases, OligoG may increase the access of therapeutic agents to bacteria and/or the surface of lung cells. In addition, OligoG can modify the structure of EPS by binding Ca^2+^ and Mg^2+^, which are involved in regulating the interaction between EPS and extracellular DNA within the biofilm scaffold [112,113]. Of note, chelation of Ca^2+^ by OligoG is related to the conformational arrangement of the G-block co-polymer [114]. Interestingly, in addition to disruption of the EPS, the potentiation by OligoG of antibiotic activity against bacteria residing in biofilms may also be mediated by the ability of this polysaccharide to modify pseudomonal motility [108]. The presence of a correlation between the inhibition of bacterial growth by OligoG and the decrease in cell motility has been previously demonstrated [115].

OligoG has been shown to modify the assembly of bacterial biofilm assembly and reduce the resistance to antimicrobial therapy [108,115,116,117]. More recently, the ability of OligoG to inhibit the growth and biofilm formation of fungi *Candida spp.* and *Aspergillus spp.* has also been demonstrated. This inhibition was associated with significantly enhanced sensitivity to antifungal agents and marked decreases in hyphal formation [21]. An essential element of the pathogenicity of *Candida albicans* is the secretion of hydrolases, such as aspartyl proteinase, phospholipase, and lipases [118], and OligoG has been shown to suppress the expression of phospholipase and secretion of aspartyl proteinases by *Candida albicans* [119].

It should be noted that the mechanisms by which OligoG suppresses the resistance of bacteria and fungi may be different. OligoG binds strongly to the cell surface of the pathogenic bacteria *Pseudomonas aeruginosa* and remains bound even under hydrodynamic shear [116,119]. In contrast, OligoG does not bind to the fungal cell wall, while still exhibiting antifungal activity. Although these studies are not directly comparable since the tested organisms were in different growth states, planktonic versus biofilm, they suggest the presence of distinct mechanisms underlying the effects of OligoG on bacterial and fungal pathogens [21].

In addition to OligoG, other forms of AOS also exhibit certain antibacterial activity. The AOS (enzymatic degradation, quantities ingested: 0.2% (w/w)) extracted from brown seaweed can alleviate the negative effects on broiler performance caused by *Salmonella enteritidis* attack and can inhibit Salmonella colonization of the cecum by promoting lactic acid bacteria (LAB) growth and modulating cytokine expression and antibody production in the mucosa [120]. In addition, AOS (enzymatic degradation, molecular weight: 4388 Kda, quantities ingested: 1 g/kg (diet)) protects shrimp against *Vibrio* infections by enhancing immune functions [121]. These findings highlight the benefits of alginate as an antibacterial agent in the breeding industry. According to some studies [107], the antibacterial activity of AOS appears to be more effective if G content is higher than 85%. On the other hand, a significant antibacterial activity against *P. aeruginosa* was observed when AOS with average sulfation degree of 6.8 were examined, while all other tested algino-oligosaccharides had little or no antibacterial activity [122]. Therefore, additional research is needed to verify the impact of G content and molecular weight on the antibacterial activity of AOS, and determine the precise molecular mechanisms of inhibition of microbial infections by OligoG.

### 2.7. Hypolipidemic Effect

Hyperlipidemia damages vascular endothelial cells, stimulates abnormal proliferation of smooth muscle cells, increases blood coagulation, and inhibits the fibrinolysis system. Together, these alterations lead to cardiovascular diseases such as thrombosis and atherosclerosis. Marine-derived AOS show significant biological activity in reducing hyperlipidemia [123]. The lipid-reducing effects of low molecular weight guluronate (molecular weight: approximately equal to 3000 Da) and sulfated low molecular weight guluronate (molecular weight: approximately equal to 3000 Da, sulfation degree: 0.85, concentration: 100 μM) were studied in a human hepatocellular carcinoma cell line, HepG2. Both compounds decreased serum levels of total cholesterol (TC) and triglycerides (TG), and inhibited the activity of 3-hydroxy-3-methylglutaryl-CoA (HMG-CoA) reductase [124]. The hypolipidemic effect of alginate was reported also in animal models [125]. Sodium alginate (quantities ingested: 50mg/kg (body weight)) administered for four weeks by gavage decreased body weight, fat accumulation, and TG and TC in high-fat diet-induced obese mice [22]. Similarly, dramatic reductions in liver cholesterol were found in rats fed with alginate, and mannuronic acid-rich alginate produced the most significant decrease in cholesterol level. The rate of cholesterol synthesis depends on the expression levels of key enzymes of the cholesterol synthetic pathway, such as HMG-CoA synthase and HMG-CoA reductase. However, the blood cholesterol-lowering effect of AOS (molecular weight: <10 KDa) depends on the inhibition of cholesterol uptake rather than on cholesterol biosynthesis [126].

The hepatic low-density lipoprotein (LDL) receptor (LDLR) has a crucial role in lipoprotein metabolism by lowering the plasma LDL-cholesterol concentration, and the decrease in LDL-cholesterol reduces the risk for cardiovascular diseases. In this regard, AOS upregulate the expression of LDLR and intracellular uptake of LDL uptake by hepatocytes, lowering plasma LDL-cholesterol levels. The upregulation of LDLR by AOS is dependent on PI3K/Akt/glycogen synthase kinase 3β (GSK3β)-mediated activation of sterol-responsive element binding protein-2 (SREBP-2), which is an essential transcription factor for LDLR gene expression. Furthermore, AOS downregulates proprotein convertase subtilisin/kexin type 9 (PCSK9), decreasing LDLR degradation in hepatocytes. Thus, AOS (concentration: 3 mg/mL) lowers the levels of plasma LDL-cholesterol by upregulating LDLR, and these effects are dependent on SREBP-2 and PCSK9 [126].

Lipid metabolism is regulated by several enzymes, including lipoprotein lipase (LPL), hepatic lipase (HL), lecithin cholesterol acyltransferase (LCAT), and cholesterol ester transfer protein (CETP). Among them, the function of LPL is of particular importance [127]. Mutations in the LPL gene or its decreased transcriptional activity lead to the development of hyperlipidemia [128]. Moreover, agents increasing the expression or activity of LPL reduce the serum levels of TC, TG, low-density lipoprotein cholesterol (LDL-C), and very low-density lipoprotein cholesterol (VLDL-C) in serum, and increase the level of HDL-cholesterol, thus reducing the incidence of hyperlipidemia and atherosclerosis [129]. In this regard, propylene glycol mannate sulfate (PGMS, molecular weight: 5000 Da, quantities ingested: 75.6 mg/kg/day) lowers the levels of TC, TG, and LDL-C, and increases that of high-density lipoprotein cholesterol (HDL-C) in the serum of hyperlipidemic rat in a dose-dependent manner. Moreover, PGMS increases the level of expression of LPL mRNA, a variable related directly to the effect of PGMS on the control of blood lipids [127].

The hypolipidemic activity of AOS and its derivatives is affected by multiple factors. Lipid accumulation in the liver of rats fed with alginate depends on the alginate viscosity, as only the high-viscosity alginate lowered lipid accumulation [130]. The composition of AOS is another relevant factor. Dramatic reductions in cholesterol were found in rats fed with polymannuronic acid, and this effect is more obvious than that of polyguluronic acid [126]. Even stronger antihyperlipidemic activity is exhibited by derivatives of AOS [124]. For example, propylene glycol alginate sodium sulfate (PSS), a sulfated derivative of alginate, has been successfully used clinically to prevent and treat hyperlipidemia and ischemic cardio-cerebrovascular diseases for the past 30 years in China [131]. Moreover, propylene glycol guluronate sulfate (PGGS), a novel sulfated polysaccharide derived from alginate, has the ability to decrease TG and TC in a dose-dependent manner. These beneficial effects are mediated by the activation of AMP-activated protein kinase (AMPK) signaling by PGGS. In view of these findings, the marine-derived PGGS merits further investigation as a novel agent for the prevention and treatment of liver-related hyperlipidemic diseases [132]. It has been also shown that amidated alginate can significantly decrease serum cholesterol, serum TG, hepatic cholesterol, and total hepatic lipids levels. In contrast, brown seaweed-derived alginate reduces significantly only hepatic cholesterol levels, but not serum cholesterol, TG, and total hepatic lipids. Thus, the hydrophobic amidated alginate is more effective as a cholesterol- and lipid-lowering agent than the hydrophilic alginate. The hypocholesterolemic effects of amidated alginate may be attributed to the increased fecal excretion of cholesterol and its derivative, coprostanol [133].

In summary, there is only a limited amount of research on the relationship between AOS structure and hypolipidemic effect but, nevertheless, the effectiveness of AOS derivatives as antihyperlipidemic agents is well-documented. The strong evidence supporting the biological activity of AOS demonstrates that AOS is a good candidate for the development of a new class of cholesterol-lowering drugs.

### 2.8. Antihypertensive Activity

A growing body of evidence shows unequivocally that alginate and AOS possess antihypertensive effects [23,24]. At least two mechanisms by which these compounds lower blood pressure have been proposed: (i) suppression of the absorption of salt in the intestine, and (ii) direct vasodilatory effect [134]. 

The classic view holds that the long-chain alginate is viscous and water-insoluble and, therefore, cannot be absorbed in the intestine and digested. Thus, it is believed that the chemical properties of alginate slow down or inhibit the absorption of cholesterol and sodium in the intestine. In this context, the carboxylic acid in alginate sugar molecules can bind Na^+^, K^+^, and Ca^2+^ cations. The ionic exchange between H^+^ and Na^+^, K^+^, or Ca^2+^ can decrease the absorption of Na^+^ in the intestine, thereby attenuating high blood pressure [24].

Recent studies have demonstrated that low molecular weight potassium alginate (average molecular weight: 1800 Da, quantities ingested: 100–500 mg/kg) can reduce blood pressure in spontaneously hypertensive rats [135], and attenuate the rise in blood pressure in deoxycorticosterone acetate (DOCA)-salt model of hypertension by a mechanism involving increased excretion of urinary sodium [136]. However, in these experiments, sodium AOS composed of only 2–3 molecules of α-l-guluronic acid and β-d-mannuronate were utilized, and the carboxylic acids were completely neutralized by Na^+^ ions during the enzymatic degradation process of alginate. These characteristics the sodium AOS employed seem to contradict the classical explanation of the mechanism of antihypertensive effects of high molecular weight alginate [134]. The possibility has been advanced that the delay or inhibition of sodium absorption is due to the presence of α-l-guluronate and β-d-mannuronate rather than the high or low molecular weight of the alginate [136].

Studies on antihypertensive effects of low molecular weight sodium AOS (degree of polymerization: 2 and 3, quantities ingested: 4% or 8% w/w (diet)) suggest that the oral administration of enzymatically degraded AOS attenuates salt-induced hypertension in Dahl salt-sensitive (Dahl S) rats in a dose-dependent fashion. The reduction in blood pressure is associated with a decrease in cardiovascular and renal damage [23]. Moreover, the antihypertensive effect of high-dose sodium alginate (quantities ingested: 8% w/w (diet) was comparable to that achieved by clinically used drugs, the diuretic indapamide, or the calcium channel blocker benidipine [137]. Moreover, the reduction in blood pressure achieved with alginate treatment was associated with a decrease in heart weight and aortic wall thickness, i.e., the typical effects of classically used antihypertensive drugs. These results suggest that the decrease in blood pressure in sodium AOS-treated rats ameliorates cardiovascular stress induced by a high-salt challenge.

Sodium AOS treatment (quantities ingested: 60 mg/day using a continuous osmotic mini-pump) via the subcutaneous route almost completely abolished salt-induced hypertension in Dahl S rats. This beneficial effect of AOS took place despite the fact that the level of fecal and urinary sodium excretion did not change significantly during the treatment. This finding indicates that the antihypertensive effects of subcutaneously administered AOS are not mediated by the inhibition or delay of sodium absorption in the intestine, but due to mechanisms other than salt metabolism [134]. Similar results were obtained in spontaneously hypertensive rats (SHR) fed a low-salt diet [138]. Of relevance, it was demonstrated that treatment with very low molecular weight sodium AOS (degree of polymerization: 2 and 3, quantities ingested: 8% w/w (diet)) attenuates spontaneous hypertension in a salt intake-independent genetic rat model mimicking human hypertension. Thus, the antihypertensive effects of sodium AOS are most likely due to its direct action on the cardiovascular system rather than inhibition of salt absorption in the intestine [134,138].

In conclusion, the mechanism of the antihypertensive effect of AOS remains to be elucidated. The exploration of sulfated derivatives of AOS (molecular weight: 4000 Da) may provide novel research concepts aiming at the elucidation of antihypertensive effects of AOS [23,134]. This possibility opens the way for new nutritional strategies aiming at the reduction of blood pressure and prevention of early-stage kidney injury.

### 2.9. Suppression of Obesity

For many people, obesity is becoming the main cause of poor quality of life by increasing the probability of metabolic syndrome, a cluster of conditions including hypertension, hyperglycemia, insulin resistance, diabetes mellitus, and hyperlipemia [139]. Several studies have demonstrated that sodium alginate and derived oligosaccharides can reduce obesity, reduce postprandial blood glucose levels, increase fat excretion, and reduce energy intake [76,140].

Numerous investigations concluded that the gut and gut flora have a vital role in the control of obesity and energy metabolism [22]. As a dietary fiber, sodium alginate can reshape gut flora, improve the function of the intestine, and regulate the metabolism of the organism maintaining body weight and preventing chronic metabolic diseases [141]. Studies of the gut transcriptome in obese mice have shown that after alginate treatment (quantities ingested: 50mg/kg (body weight)), differentially expressed genes are involved in lipid metabolism and carbohydrate metabolism. The alteration in the gut transcriptome indicates that sodium alginate can effectively inhibit obesity and obesity-associated metabolic syndrome [22].

A fiber-rich diet is widely believed to be associated with the low prevalence of obesity [142,143]. As with other types of dietary fiber, sodium alginate can counteract obesity by regulating human appetite, and the gelation of alginate in the stomach appears to be critical for this effect [123]. For example, consumption of a large volume of sodium alginate gel drink (325 mL sweetened, milk-based meal including 1% by weight alginate) is associated with stronger postprandial satiety [144]. In addition, postprandial glycemic response is attenuated following the consumption of an alginate-based glucose beverage [145,146]. Sodium alginate has also been shown to reduce intestinal absorption of cholesterol and glucose in several animal models [147,148].

The ability of sodium alginate to affect a number of physiological processes controlling food intake is related to its ability to viscosify and form a gel within the gastrointestinal tract [123]. Alginate may suppress glucose absorption by reducing gastric emptying and nutrient absorption due to the viscosification of gastric contents [145,146]. Although the mechanisms through which fiber-rich diet modulates food intake are not completely understood, their low energy density, hypolipidemic properties, ability to maintain glycemic homeostasis, capacity to delay nutrient absorption, and the potential to cause gastric distension and improve satiety appear to be essential [149].

A property of alginate that may be important for its potential to modify eating behavior is its ability to gel upon contact with multivalent cations [149]. This characteristic is more pronounced in alginates rich in G-blocks which form the strongest gels due to a spatial conformation favoring ionic cross-linking [150]. These findings suggest that strongly gelling alginate formulations should be developed to manage the ever-increasing problem of overweightness and obesity.

### 2.10. Hypoglycemia

Diabetes mellitus is the most common metabolic disease and its prevalence is increasing in both developed and developing countries [151]. In recent years, a significant number of different poly- and oligosaccharides have been investigated and their antidiabetic activity has been demonstrated [25]. Among them, AOS and sulfated AOS exhibit potent hypoglycemic effects [22]. Marine-derived polysaccharides stimulate insulin secretion in vitro, particularly low molecular weight (molecular weight: approximately 3 kDa, concentration: 100 μM) oligosaccharides [25]. In this respect, polyguluronate sulfate (PGS, concentration: 20 μg/mL) and its oligosaccharides are excellent enhancers of FGF1/FGFR1c and FGF19/FGFR1c signaling and merit further investigation as novel agents for the treatment of type 2 diabetes [152]. Additionally, oligomannuronate and its chromium (III) complexes (molecular weight: 3 kDa, concentration: 50 μM) improve insulin sensitivity in the C2C12 line of skeletal muscle cells, and act as a novel glucose uptake stimulator with low toxicity. Considering this property, these compounds have the potential to be used as a dietary supplement or drug for the treatment of type 2 diabetes mellitus. Mechanistically, the improvement in insulin sensitivity may involve the upregulation of the expression of insulin receptor and glucose transporter 4 (GLUT4) mediated by the activation of both insulin (PI3K/Akt) [153] and AMPK [154] signaling pathways in the skeletal muscle. Moreover, oligosaccharides (molecular weight: 3 kDa, concentration: 50 μM) localize in C2C12 cells to the mitochondria and increase the expression of PPARγ co-activator (PGC)-1α, indicating that their actions may be associated with the functions of mitochondria [151].

As a dietary fiber, the AOS and oligomannuronate-chromium complex may be used as adjuvant therapy in type 2 diabetes. These compounds could enhance insulin sensitivity with a toxicity profile lower than that of commonly used metformin.

### 2.11. Promotion of Cell Proliferation

AOS can stimulate mammalian cell proliferation, particularly in the presence of growth factors [155]. Human keratinocytes are epithelial cells that can synthesize keratin, which can act as a barrier in the body and protect the human body. Promoting the proliferation of human keratinocytes and extending their lifespan have important clinical significance [156,157,158]. AOS (enzymatic degradation, concentration: 10 mg/mL) cleaved from alginic acid polysaccharides stimulates the proliferation of keratinocytes in the presence of the non-heparin-binding epidermal growth factor (EGF) [159]. These results will provide important clues for improving human keratinocyte culture conditions, which may help to quickly obtain a large number of normal human keratinocytes in vitro in the future, and lay a good theory and reality for basic research and clinical application of human keratinocytes basis. Similarly, AOS (enzymatic degradation, concentration: 5 µg/mL) can activate multiplication and migration of endothelial HUVEC cells in the presence of recombinant VEGF165; in fact, endothelial cells may be more susceptible to the stimulation by AOS keratinocytes [155]. The analysis of the structure-activity relationship indicated that AOS possessing guluronic acid in the terminus are better activators of cell proliferation in the presence of VEGF165. Peripheral guluronic acid may be effective because of an affinity for the receptor on endothelial cells or VEGF and may cause more enhanced stimulation. However, the exact relationship between the ability of alginic acid oligosaccharides to promote proliferation and their chemical structure is unknown at present [155,159].

### 2.12. Regulate Plant Growth

While there are few studies on the promotion of proliferation of animal cells by AOS (enzymatic degradation, degree of polymerization: 2–4), its effects on plant cell growth are more pronounced and are more manifested in regulating plant growth, such as the green alga Chlamydomonas reinhardtii and a Unicellular Marine Microalga, Nannochloropsis oculata [26,160]. ADO (enzymatic degradation, molecular weight: 1445 Da, concentration: 0.75 ‰ (v/v)) can enhance the germination of plant seeds, most likely by promoting amylase activity and accelerating their metabolic activity [161]. In addition, AOS at 10–50 mg/L increases the root length, the number of root tips, root volume and fresh weight, root absorption area in flowering Chinese cabbage (*Brassica campestris* L.), and, under the same culture conditions, the impact of AOS was greater than with other oligosaccharides such as oligogalacturonic acid [162]. These effects may be one of the main mechanisms by which AOS improves crop growth and yield [163].

The root system is essential for providing the area for absorption of water and other nutrients by plants, as well as anchoring them in the soil [164]. Growing evidence supports the notion that AOS promote the growth of plant roots. For example, AOS (degree of polymerization: 3–6) obtained by enzymatic hydrolysis produces an approximately 2-fold enhancement in the root growth of lettuce at concentrations ranging from 200–3000 μg/mL [165]. Similarly, the enzymatically digested mixture of polyguluronic acid (degree of polymerization: 3–9) promotes the growth of roots in carrots and rice (*Oryza sativa* L.), with degree of polymerization 5 (DP 5, concentration: 0.75mg/mL) having the highest activity [163]. These growth-stimulating effects may be due to the direct or indirect effects of AOS on the auxin signaling. AOS (degree of polymerization 2–4, average molecular weight: 724 Da, concentration: 10–80 mg/L) induces in a dose-dependent manner the expression of auxin-related genes OsYUCCA1, OsYUCCA5, OsIAA11, and OsPIN1 in rice tissues, accelerating the biosynthesis and transport of auxin, and reduces the activity of indole-3-acetic acid (IAA) oxidase in rice roots. These changes resulted in a 37.8% increase in IAA concentration in rice roots, upregulation of genes controlling root development, and enhanced root growth [164]. Additionally, AOS (enzymatic degradation, degree of polymerization: 2–4, concentration: 10–80 mg/L) can induce the synthesis of NO, promoting root growth in the wheat [166]; however, the mechanism has not been identified yet. NO interferes with auxin signaling through *S*-nitrosylation of the *Arabidopsis* TRANSPORT INHIBITOR RESPONSE 1 auxin receptor [167], and this finding may suggest novel mechanisms of promotion of root growth by AOS.

The degradation mode and molecular weight have a significant effect on AOS-induced plant growth. The growth of *Chlamydomonas. reinhardtii* and cellular fatty acid content is significantly increased by an AOS prepared by enzymatic degradation. However, AOS (acid hydrolysis) and other saccharides do not affect the growth of the alga. Differences in molecular structures might account for the differences in the effects of AOS (enzymatic degradation) and AOS (acid hydrolysis). For example, the unsaturated terminal structure with a double bond in AOS (enzymatic degradation) may be linked to its bioactivity. In addition, the differences in the effects of AOS (enzymatic degradation) and AOS (acid hydrolysis) on the stem of *Chlamydomonas. reinhardtii* may also result from different molecular sizes contained in the mixtures [160]. AOS with degree of polymerization between 2 and 10 can increase the yield of crops, such as Carrot, Rice Plants and Brassica chinensis. [162,163]. To gain insight into the structure-activity relationship in alginate oligomers, it is crucial to use highly purified samples. Therefore, it will be necessary to purify various oligomers with different degrees of polymerization and analyze separately their effects.

In summary, AOS may be used as a new type of plant growth regulator. This application would represent a novel and very significant utilization of marine resources in agriculture. However, progress in this area is currently limited due to the lack of a clear understanding of the mechanism of the action of AOS on plant cells [164]. 

### 2.13. Other Activities

In addition to the above-discussed biological activities of alginates and their degradation products, other aspects of their unique effects have also been reported. For example, AOS (molecular weight: 10 kDa) produced by *Bacillus subtilis* strain KCTC 11782BP was shown to effectively suppress asthma via T-helper cell type 2-related cytokines [168]. AOS used as a dietary supplement has also been reported to beneficially affect the growth of weaned pigs, most likely by improving intestinal morphology and barrier function. In the same application, AOS inhibited mitochondria-dependent apoptosis of enterocytes [52]. In research related to human ageing, guluronic acid restored the expression of the superoxide dismutase 2, glutathione peroxidase 1, catalase, glutathione *S*-transferase, iNOS, and myeloperoxidase genes to normal levels, indicating that it might reduce the age-related changes [169]. PGS, a sulfated derivative of polyguluronate, exhibits good biocompatibility and has structural properties similar to those of heparin [170]. PGS has excellent prospects for clinical applications as an agent protecting from thrombi formation, inflammation, urinary calculi, autoimmune liver damage, and viruses [170,171,172,173,174,175]. These activities of AOS expand the possibilities for their pharmacologic use.

## 3. Concluding Remarks and Future Outlooks

Marine-derived alginate and its oligosaccharides exhibit an extraordinary variety of biological activities, providing new avenues for the treatment of human diseases. The recent development of the sodium oligomannate GV-971 gave new hope to Alzheimer’s disease patients, and, at the same time, boosted the confidence of scientists that additional profound developments in the utilization of active substances of marine origin are yet to be discovered.

It should be noted that the diversity of the biological properties of alginate and its degradation products are closely related to their structural diversity. This association prompted studies of multiple essential factors that modulate the biological activity, including degradation patterns, molecular weight, G content, spatial conformation, and its derivatives; for example, the sulfated derivatives of AOS and the polyalcoholized mannoglucan. In studies of the biological activity of alginate, the appropriate degradation method should be selected at first. The AOS obtained using the common methods of acid, oxidative, and enzymatic degradation display significant structural differences. As an example, a carboxyl group is present at the end of the alginate oligosaccharide obtained by oxidative degradation, while a conjugated olefin acid structure exists in the AOS obtained by enzymatic hydrolysis. These specific structures affect biological activities of AOS, with acid hydrolysis yielding a product with lower biological activity. In addition, single factors alone do not determine the activity of AOS, which represents an outcome of superimposed effects of many factors. The spatial conformation of AOS does have an important effect on its activity, but is, in turn, by the molecular weight and G content of the molecule. Thus, the impact of AOS conformation on its activity can be restricted by several independent factors. Moreover, alginate oligosaccharide derivatives also exhibit excellent biological activity. For example, the polyguluronate sulfate discussed in this review has a satisfactory hypolipidemic effect, and the sulfated derivatives of AOS have more potent antitumor activity non-modified AOS. These results, indicating superior biological activity of derivatized AOS, prompted further research in this field. Thus far, studies on the structure-activity relationship of alginate and its degradation products are still limited, and most investigations focus only on the activity of AOS mixtures. Therefore, despite inherent difficulties, it is critical to obtain the best possible purity of the sample when studying the structure-activity relationship of alginate oligomers.

In conclusion, although alginate degradation products exhibit a wide range of biological activities, there are still only a few products that can be used clinically. Detailed pharmacological and pharmacokinetic studies of AOS emerge as another challenge in the area of alginate research.
